# Long-Term Phytoplankton Dynamics in a Complex Temporal Realm

**DOI:** 10.1038/s41598-019-52333-z

**Published:** 2019-11-04

**Authors:** M. Alvarez-Cobelas, C. Rojo, J. Benavent-Corai

**Affiliations:** 1 National Museum of Natural History (CSIC), c/ Serrano 115 dpdo, Madrid, E-28006 Spain; 2 Cavanilles Institute of Biodiversity and Evolutionary Biology, University of Valencia, c/ Catedrático José Beltrán 2, Paterna, Valencia, E-46980 Spain

**Keywords:** Freshwater ecology, Limnology

## Abstract

Faced with an environment of accelerated change, the long-term dynamics of biotic communities can be approached to build a consistent and causal picture of the communities’ life. We have undertaken a 25-year monthly-sampling study on the phytoplankton of a meso-oligotrophic lake, paying attention to controlling factors of overall biomass (TB) and taxonomical group biomass (TGBs). Long-term series included decreased trends of TB and TGBs, and multi-scale periodicity. A decadal TB periodicity emerged related to nitrogen concentration and Cryptophytes. Annual periodicities were mainly related to air and water temperature controlling the abundance of Chlorophytes or Dinoflagellates. Intra-annual cycles could arise from autogenic processes. The analysis by periods revealed relevant dynamics (for example, Diatom periodicities), hidden in the analysis of the complete series. These results allow us to establish that: i) two organizational levels of phytoplankton change differently in time scales from months to decades; ii) controlling factors (climate, water physics and chemistry) act at different time scales and on different TGBs, and iii) different combinations of the “taxonomical group-control factor-trend and periodicity” set throughout the studied time explain total biomass dynamics. A holistic approach (multiple complementary analyses) is necessary to disentangle the different actors and relationships that explain non-stationary long-term phytoplankton dynamics.

## Introduction

Faced with an environment of accelerated change^1^, the long-term dynamics of biotic communities can be approached to build a long-term, consistent and causal picture of the communities’ life. They often respond to instability, and this fact makes it necessary to disentangle the time scales involved to predict their future behaviour.

When trying to understand long-term ecosystem dynamics, we are confronted with several questions and decisions. How can we identify trends and periodicities in the dependent signals? Do they have any ecological meaning? What are the controlling factors of variable dynamics? Do we have to study ecosystem features (such as biomass), or do we have to deal with other levels of biological complexity (such as groups of species)? Would the controlling factors be the same across all levels of organization? Would they be the same at all temporal scales? Are the responses the same throughout the whole period of study?

Furthermore, the temporal length of observations along with their high frequency is important to cover all modes of variability. Some phytoplankton studies have reported decadal periodicities^2,3^. Hence time spans much longer than 10 years are needed to suggest an insightful picture of organismic responses and their controlling factors, providing that the sampling frequency is high enough to disclose some parts of community effects. The idea of multiple scales (from seconds to decades) of responses by different phytoplankton features (from physiological rates to community composition and biomass) arose for the first time at the beginning of the eighties^4^. We therefore focus our approach and discussion on studies longer than one decade that encompass a sampling frequency to cover most community effects.

Recently, there have been many studies dealing with long-term freshwater phytoplankton dynamics, addressing parts of those aforementioned questions. Some have usually considered biomass, or its chlorophyll-a surrogate, as the only signal. Table 1 compiles studies longer than 10 years of observations with frequencies of observation (monthly or shorter) which are useful to detect modes of community variability. A variety of suggested causes for the trajectory of long-term (>10 years) phytoplankton biomass exists (Table 1). For example, they include the often expected covariation with phosphorus concentration^5,6^ which is considered the most likely cause to explain phytoplankton changes in addition to others, such as the intended-or-not species introduction (*e.g*. mussels^7^), the exclusion of food-web top species (*e.g*. cyprinid fish^8^) and the impact of fungal epidemics^9^ and/or atmospheric depositions^10^.

**Table 1:** Long-term responses of freshwater phytoplankton biomass and their causes and periodicities in studies longer than 10 years with frequent sampling (BW: biweekly, M: monthly, W: weekly).

Lake	Latitude	Period and frequency of study	Causes (linked or not)	Phytoplanktonic biomass response	Reference
Balaton	47 °N	1980–2002 (M)	P decrease, Cyprinid elimination	Decrease	^8^
Biwa (Northern basin)	35 °N	1962–2003 (BW)	Eutrophication until 1985, Global warming	Earlier increase, Later decrease	^12^
Biwa (Northern basin)	35 °N	1979–2009 (BW)	Global warming, Wind increase, PAR limitation	Earlier increase, Later decrease	^44^
Crater	43 °N	1984–2000	Global warming, Atmospheric deposition increase, P increase	Increase	^45^
Garda	46 °N	1993–2009 (every 4^th^ week)	P increase, EA decrease	Increase	^15^
Geneva	47 °N	1975–2010 (M or BW)	P decrease, *Daphnia* impact	Fluctuating (higher variability at the extremes of the series)	^46^
Grasmere	54 °N	1970–2010 (W or BW)	Water renewal increase, P decrease	Decrease	^47^
Greifensee	47 °N	1971–2000 (BW or M)	P decrease	Fluctuating (high variability)	^18^
Heiligensee	53 °N	1975–1992 (BW or M)	P increase	Increase	^5^
Kinneret	33 °N	1969–2003 (W or BW)	N limitation, fungal epidemics	Increase	^9^
Krankesjön	56 °N	1980–2011 (BW or M)	Global warming, Nutrient availability, Food web	Fluctuating summer biomass	^48^
Loch Leven	56 °N	1968–1985 (W, BW or M)	P decrease	Decrease	^49^
Luzern	47 °N	1955–2000 (W, BW or M)	P decrease	Decrease	^6^
Maggiore	46 °N	1984–2005 (BW or M)	P decrease	Decrease	^50^
Müggelsee	52 °N	1979–2003 (W or BW)	P decrease	Decrease	^51^
Neusiedlersee	48 °N	1968–2007	NAO increase	Winter increase	^52^
North Pine Dam	27 °S	1978–1994 (W)	Fluctuating water renewal	Fluctuating (high variability)	^53^
Ontario (Bay of Quinte)	44 °N	1972–2008 (BW or M)	P decrease	Decrease	^54^
Ontario (main lake)	44 °N	1975–2000	P decrease	Decrease	^7^
Ontario (main lake)	44 °N	2001–2012	P increase, Global warming, *Dreissena* increase	Fluctuating	^7^
Orta	46 °N	1984–2008	P decrease	Decrease	^15^
Pyhäjärvi	62 °N	1963–2002 (BW)	P decrease	Decrease	^55^
Saidenbach	50 °N	1975–2011 (W or BW)	P decrease, global warming	Increase	^10^
Vänern	59 °N	1980–2012	P decrease, Atmospheric deposition decrease, Global warming	Increase	^13^
Washington	47 °N	1975–1999 (W or M)	ENSO increase	Winter and spring increase	^14^
Windermere (North basin)	54 °N	1850–2000	NAO increase	Increase	^56^

EA: Eastern Atlantic Oscillation, ENSO: El Niño-Southern Oscillation, N: nitrogen, NAO: Northern Atlantic Oscillation, P: phosphorus.

One of the main factors believed to act on long-term phytoplankton dynamics is global warming, but its effects are not always as expected^11^. For example, phytoplankton biomass has been shown to decrease with global warming^12^, or in other instances its effects are counterbalanced by other proccesses such as reoligotrophication, resulting in resilient phytoplankton structures^10,13^. There are some studies (Table 1) that deal with long-term biomass responses to local environments, including local warming as one of the drivers^5,8^. Others also try to link global warming and phytoplankton, using linear relationships of the latter with water temperature to prove this^10,13^. Many studies have looked for teleconnections^11^ (*i.e*. regional climate) as factors controlling long-term phytoplankton dynamics, reporting data gathered from frequent sampling (monthly or shorter^14,15^).

Furthermore, at the organizational level there is a missing link. The dynamics of total biomass is currently explained by species replacement over time driven by changes in local conditions, which often imply a replacement of taxonomic groups^16^. Previous studies have suggested that these groups can sometimes prove to be better indicators of environmental conditions and effects than species-specific data^17^. In addition, changes at this class-level of taxonomic composition are usually a good reflection of seasonal changes in the system^16^, as well as its interannual pattern. Despite this, taxonomical groups have not been considered very often when addressing longer-term (>10 years) phytoplankon changes (but see, for example, studies on lakes Greifensee^18^, Tahoe^19^ and Zürich^20^).

A further topic worth mentioning in long-term ecological studies is the development of statistical methodologies to deal with time series data. Until recently, spectral decomposition of long-term series (detrended series and detection of periodicities)^21^, and general linear models to disclose the long-term relationship between a dependent biotic signal and an independent environmental factor^22^, have been the preferred methodologies. These approaches assume that the series is stationary, *i.e*. constant variance over time, but such an assumption has been refuted recently for phytoplankton^23^. In addition, both approaches do not provide information on how much variability is explained by trend and time scales of the signal, and the controlling factors involved.

Over the last two decades, some methods have been implemented to improve and complement the classical approach (*i.e*. spectral decomposition), thus enabling to quantify the relative importance of patterns and processes across time scales of ecosystems, namely, to partition variability at different temporal scales^24^. The first procedure, called Asymmetric Eigenvectors Maps (AEM hereafter), summarizes temporal scales on a set of vectors that can be used as covariates when modelling ecological responses to environmental variability over time^25^. The second, known as variance partitioning, has been developed to quantify the individual contribution of temporal scales and environmental factors overcoming the collinearity problem^26^. A third method (codependence analysis) allows the main factor associated with each temporal scale^27^ to be identified. These methods consider the studied time series as stationary. Notwithstanding this, ecological communities can be variable through time, and a fourth method, wavelet analysis, enables the emergence and/or disappearance of periodicities over the whole series to be identified^28^. These methodologies have already been applied successfully to processes in freshwater ecosystems^29,30^, and usually result in a better description of temporal scales. However, very few studies (*e.g*.^31^) are available using all these novel methods to characterize long-term phytoplankton processes, periodicities and controlling factors, as we advocate here.

Our hypotheses to be tested here are the following: 1^st^) the total biomass of phytoplankton and those of its taxonomical groups show different trends and periodicities that change along the whole time series; 2^nd^) taxonomical composition varies over time exhibiting periodicities longer than seasonal; 3^rd^) trends and periodicities of both phytoplankton biomass and taxonomical groups biomass depend on the time period, and 4^th^) there could be different controlling variables of total- and taxonomical groups’ biomass in different time periods and at different temporal scales. Therefore, we expect that the response at specific time scales of each taxonomical group to environmental factors at each time period could provide insights into phytoplankton biomass dynamics.

Using 25 years of monthly data on phytoplankton, and its likely controlling factors in an oligo-mesotrophic lake, we aim to address all these issues. We will deal with two levels of organization (overall biomass and taxonomical composition), testing the occurrence of multiple scales of change (from months to decades) and quantifying the explicative value of controlling factors (either regional or local) of phytoplankton dynamics. Our study will demonstrate that there are different trends, periodicities and controlling factors for different levels of organization, and that there can be discontinuities and multiscale changes in long-term phytoplankton dynamics, too.

## Results

### Long-term trends and periodicities

**Figure 1:**
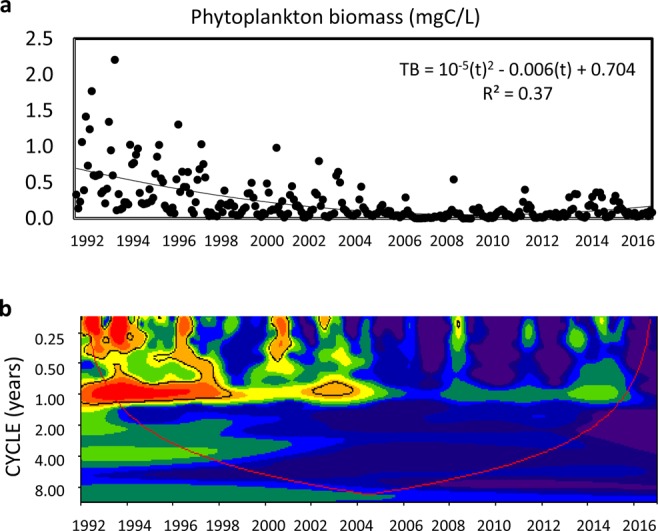
(**a**) Water column-averaged, monthly phytoplankton biomass in Las Madres lake from January 1992 to December 2016. The series is fitted to a non-linear function, which is also shown along with its statistically significant explained variability (R^2^, P < 0.001). (**b**) Continuous wavelet power spectrum showing the periodicity of monthly phytoplankton biomass in Las Madres lake. The thick black contour delimits the significant periodicities (P < 0.05) and the red line denotes the cone of influence, where edge effects may distort the interpretation. Colors reflect the strength of intensity or power (dark red indicates high power; dark blue indicates low power).

Total phytoplankton biomass (TB hereafter) experienced a decreasing trend in Las Madres lake for the whole period under study (Fig. 1a). The analysis of time series using AEM revealed a negative trend for TB from 1992–2016 (Table 2), as already mentioned, and two further periodicities: a decadal (17 years) and an annual one (Table 2). Wavelet analysis also enabled us to visualize an intra-annual periodicity at least in the first quarter of the studied series (Fig. 1b). Moreover, this analysis was able to identify the temporal variability in the periodicity of TB, as shown by a remarkable annual periodicity up to 1998, later weakening until 2006 when it disappeared (Fig. 1b).

**Table 2:** Adjusted variance (%R^2^_adj_) of biomass models in Las Madres lake in different periods shown when statistically significant (P < 0.05) (see also text and Figs 1, 2).

	TB	CHLOR	CRYP	DIAT	DINO
**1992–2016**					
Trend	(−)25	(−)9	(−)20		(−)16
Cycle in months (years)					
201 (17)	13		17		
12 (1)	18	15			39
6 (0.5)					6
**1992–1998**					
Trend	(−)9		(−)11		
Cycle in months (years)					
12 (1)	37	27	10		44
6 (0.5)		11			13
**1998–2006**					
Trend					(−)6
Cycle in months (years)					
12 (1)	26	11		14	29
6 (0.5)				8	6
**2006–2016**					
Trend	(+)8	(−)9		(+)12	
Cycle in months (years)					
12 (1)	21		16		20

Asymmetric Eigenvector analysis plus forward selection with two stopping criteria were used (see Supplementary Methods).

Trends are shown with the sign of the relationship in brackets. TB is overall phytoplankton biomass. CHLOR is Chlorophyte biomass, CRYP – Cryptophyte biomass, DIAT – Diatom biomass, DINO – Dinoflagellate biomass.

Throughout the whole series (1992–2016), all biomasses of taxonomical groups (TGBs hereafter), except that of Diatoms, showed a trend and periodicity in their dynamics (Table 2). Chlorophytes and Dinoflagellates had annual cycles and the latter also exhibited an intra-annual periodicity; Cryptophytes had interannual cycle (Table 2). Wavelet analysis complemented this information by highlighting that: i) annual periodicities took place in the early period, ii) Diatom dynamics had weaker annual periodicity over the whole 25-year period, and iii) Cryptophyte periodicity vanished after 1998 (see Supplementary Fig. S1).

**Figure 2:**
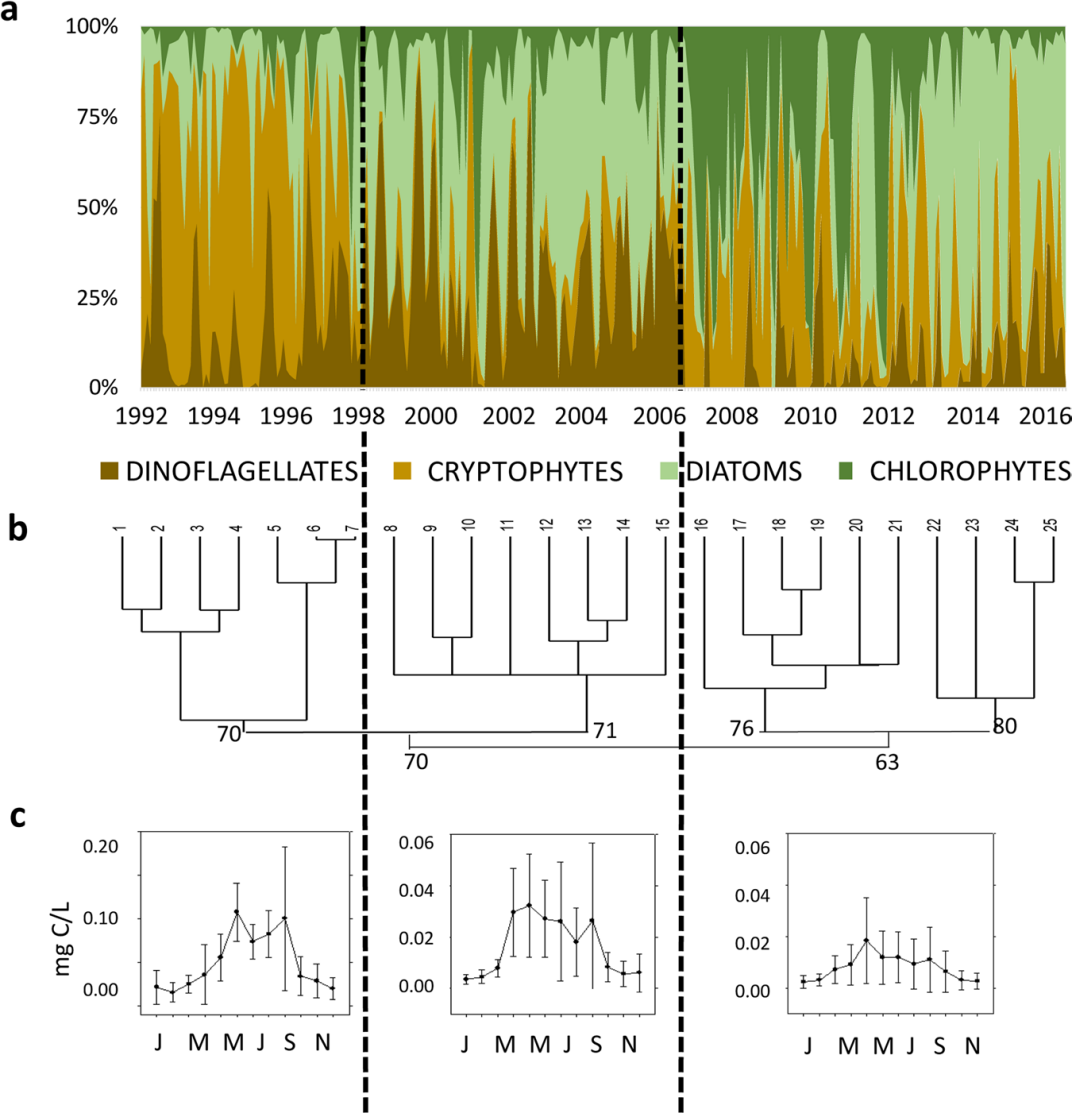
(**a**) Changes in the structure of taxonomical group biomass (TGBs) in Las Madres lake from January 1992 to December 2016. (**b**) Dendrogram of yearly-averaged biomass (%) of taxonomical groups; the main bootstrap values have been included. (**c**) Average seasonality of total phytoplankton biomass in the intervals reported. Notice the changes of scale between the early interval and the other two, latter ones. Vertical dashed lines separate different temporal intervals.

TGBs encompassed the greatest overall biomass over the study period. Cryptophytes dominated during the early years of the series, and two decades later they were co-dominant with Chlorophytes. Diatoms were present throughout the series, whereas Dinoflagellate biomass seemed, sometimes, to be negatively correlated with that of Cryptophytes (Fig. 2a). These changes in TGB composition enabled us to establish three periods of phytoplankton dynamics (Fig. 2a), which were well defined by a cluster analysis (Fig. 2b).

The first group of dates occurred from January 1992 to April 1998 (76 months), with TB amounting to 0.50 ± 0.43 mg C/L, and this was followed by the period of May 1998-June 2006 (96 months), with 0.17 ± 0.18 mg C/L, and that of July 2006-December 2016 (126 months), with 0.08 ± 0.09 mg C/L. Thus, the average TB in each interval was less than half that of the previous period. TB peaks were also different in these three periods (Fig. 2c), and this resulted from increasing their CVs (first period: 86%; second period: 106%; third period: 113%). In the earlier one, there were two peaks along with a higher monthly variability. The autumnal peak showed a gradual decline, as shown in the second and the third intervals; in the latter monthly variability was also lower than in the second period.

**Figure 3:**
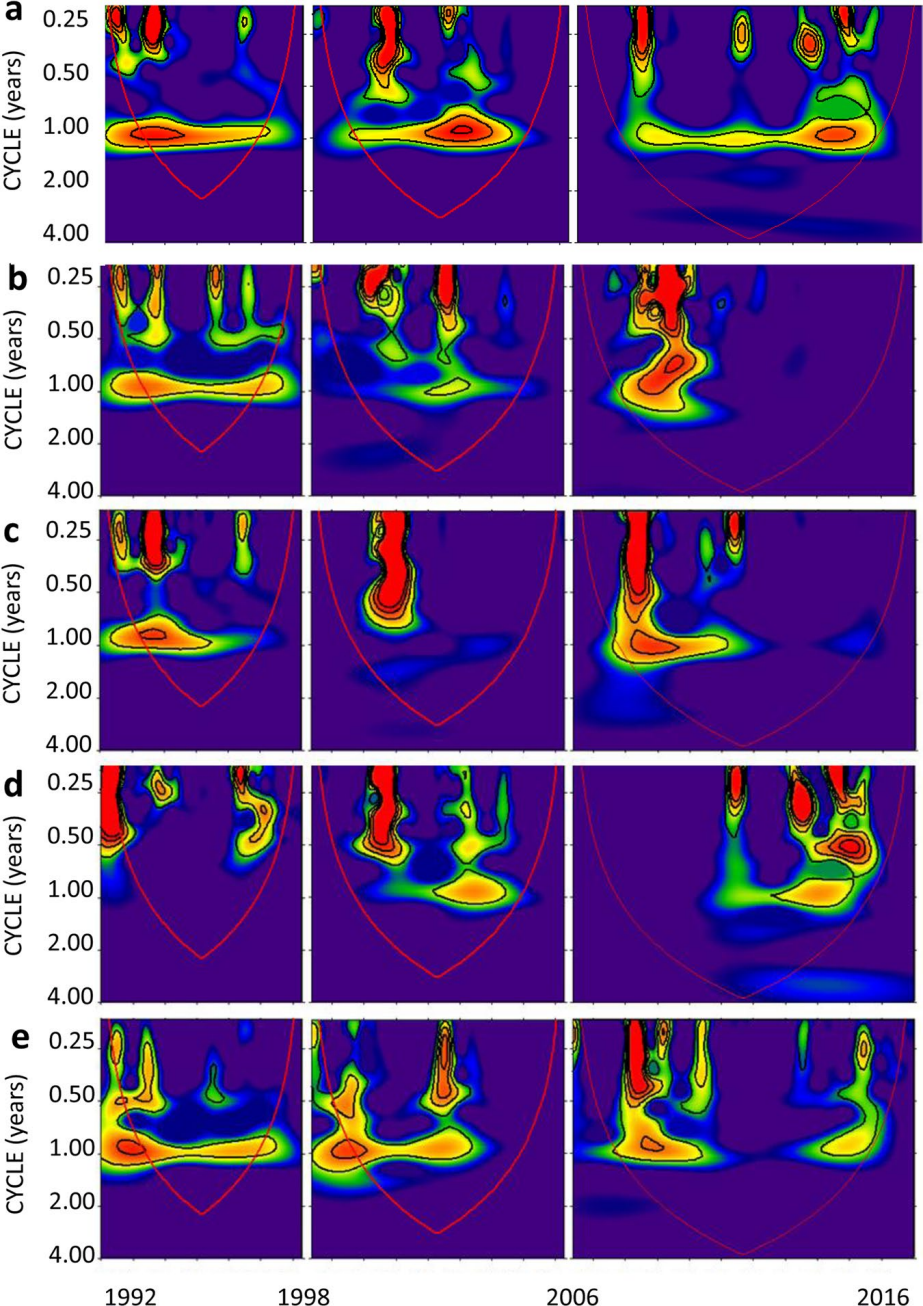
Wavelet power spectra showing periodicities from the three reported intervals in Las Madres lake between 1992 and 2016 (see Fig. 1). (**a**) Total phytoplankton biomass, (**b**) Chlorophyte fractional biomass, (**c**) Cryptophyte fractional biomass, (**d**) Diatom fractional biomass, and (**e**) Dinoflagellate fractional biomass. Explanations as in Fig. 1b.

In order to uncover likely periodicities masked by uneven fluctuations over the whole series (1992–2016), trend and periodicity were analyzed in each of the three aforementioned periods. TB dynamics followed a negative trend until 1998, later lacking any trend until 2006 and weakly increasing henceforth up to 2016 (Table 2, Fig. 1a). TB only experienced an annual periodicity and, as the wavelet analysis suggested, more variance was explained in the first period than in the remaining ones (Table 2, Fig. 3).

In the first period, only Cryptophyte biomass showed a significant decreasing trend, whereas the lowest biomass variability was experienced by Dinoflagellates. Chlorophytes and Diatoms showed different trends, declining and increasing respectively (Table 2, Fig. 3). The only group with an annual periodicity in the three intervals was Dinoflagellates, which also exhibited intra-annual periodicity during the two earlier intervals. Such an annual periodicity became less important over time (Table 2, Fig. 3), a fact matching an ongoing smoothing of the intra-annual bimodal pattern in the second period, and its waning in the third one (Fig. 2c). Chlorophyte contribution to overall biomass also varied among periods, with intra or annual periodicities occurring only during the first two intervals. The annual periodicity of Cryptophytes was lost during the second interval, which was the only period when Diatoms showed any periodicity (Table 2, Fig. 3).

### Controlling factors

**Figure 4:**
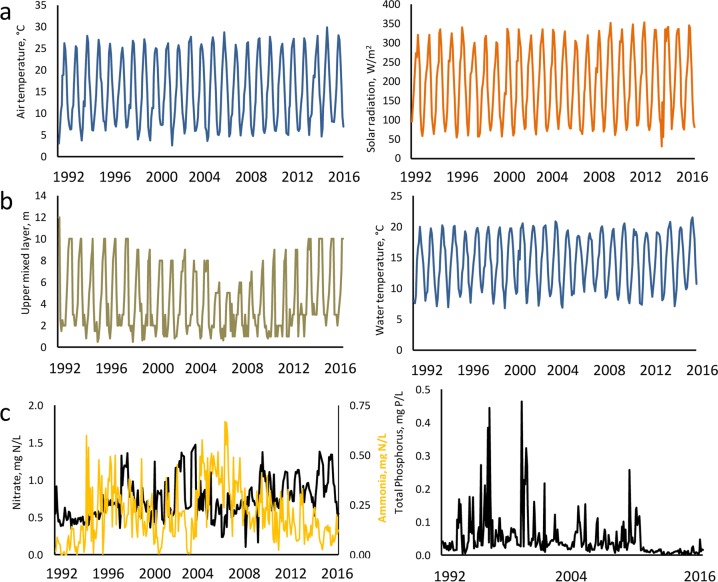
Monthly records of some relevant abiotic variables of (**a**) local climate, (**b**) water physics and (**c**) water chemistry in Las Madres lake from 1992 to 2016. Lake variables are water-column averages. The first data is January 1992, the last one is December 2016.

Regional climate teleconnections did not explain any variability for the overall period. Local climate variables, such as air temperature and solar radiation (Fig. 4a), had a positive relationship with TB dynamics (Table 3), the local factor explaining a 17% of TB variability. Water temperature and the upper mixed layer depth (physical factors, Fig. 4b) explained 15% of overall variability at most (Table 3). Nitrate and ammonia (chemical factors, Fig. 4c) only explained 7% (Table 3).

**Table 3:** Control variables that explained more than 5% of total phytoplankton biomass or taxonomical group biomass with the sign of their relation and their percentage of explained variance (R2).

1992–2016	VARIABLES R2 >5%			MODELS		
Response variable	Sign	Control variable	R2	R^2^adj of selected variable	Environmental factor	R^2^adj
Total Biomass	+	Air temperature	15		LOC	17
	+	Radiation	17	17 (a)		
	−	Zm	7		PHY	15
	+	Water temperature	15	15 (a)		
	−	Nitrate	5	5 (d)	CHE	7
	−	Ammonia	5	4 (d)		
Chlorophyte biomass	+	Air temperature	12	12 (a)	LOC	12
	+	Radiation	9			
	+	Water temperature	11	11 (a)	PHY	11
Cryptophyte biomass	−	Nitrate	5	5(d)	CHE	5
Dinoflagellate biomass	+	Air temperature	17		LOC	18
	+	Radiation	18	18 (a)		
	−	Zm	11	1 (a)	PHY	16
	+	Water temperature	15	15 (a)		

Also included, their adjusted explained variance (%R^2^_adj_) if selected by forward selection procedure and the adjusted explained variance of the individual effects of factors (last column), without interactions. This last value results from the RDA and variance partition analysis. Analysis performed for the whole study period (1992–2016) in Las Madres lake. Probability of all variances was lower than 0.001. For more information on statistical tools see Supplementary methods.

Diatom biomass was not related to any analysed factor. The “a” letter implies annual co-dependence whereas “d” is decadal co-dependence (see Table S1). While TB is overall phytoplankton biomass, Zm is the thickness of the upper mixed layer. CHE: water chemistry, LOC: local climate, PHY: water physics.

With regard to warming in Las Madres lake, we did not observe this using over 25 years of monthly-averaged air temperature data. However, an increase in the annual average temperature (see Supplementary Fig. S2a) shows an increase of almost 1 °C/decade in air and 0.75 °C/decade in water. The annual average of the upper mixed layer experienced a decreasing trend until 2010, after which it increased again (see Supplementary Fig. S2b).

Chlorophyte biomass was mainly related to radiation and air- and water temperature, hence being related to local factors such as climate and water physics. Cryptophyte dynamics was weakly explained by water chemistry, with an inverse pattern to that of nitrate concentration. Dinoflagellate dynamics was explained by local climate, but also by some physical properties of the water column, such as temperature and mixing depth (Table 3).

**Figure 5:**
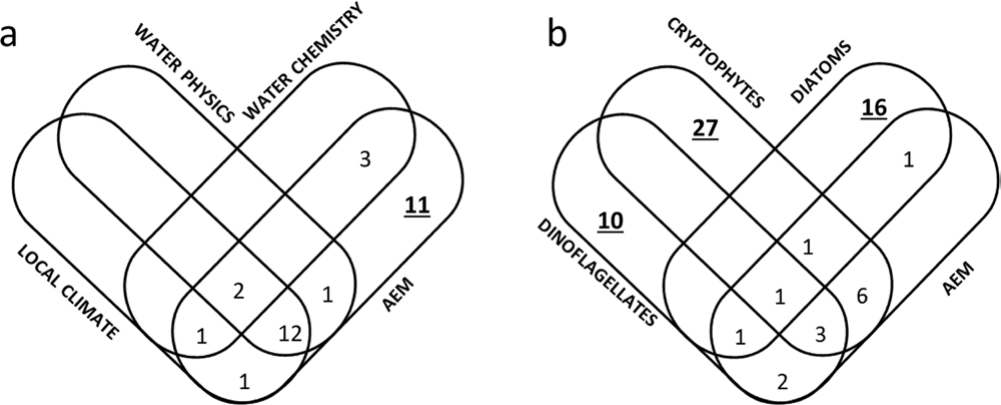
(**a**) Variance partitioning of a model with overall phytoplankton biomass as the dependent variable and local climate, physical and chemical proprieties of water column and AEM (periodicities) as controlling factors in Las Madres lake for 1992–2016. (**b**) The same for the biomass of taxonomical groups. Only variance related to pure effect of controlling factors can be tested, being its value in bold and underlined lettering when statistically significant (P < 0.05). Only non null variances for both pure and interaction effects are shown to make the figure clearer. For the same reason, Chlorophytes are neglected because their pure adjusted variance was lower than 2%, in spite of the fact that their interaction with Cryptophytes amounted to 15% of explained variability.

The partition of variance in the model TB = [LOC] + [PHY] + [CHE] + [AEM], where environmental factors only included abiotic selected variables (those mentioned in Table 3), showed that TB variability was explained by interactions between abiotic factors and AEM periodicities (annual and decadal), amounting to 20% of overall variability and pure AEM (Fig. 5a). The relevant interaction with periodicity was also demonstrated by multiple co-dependence analysis (MCA; see Supplementary Table S2), which revealed that variables of local climate were the most influential predictors of TB along with annual periodicity, whereas water chemistry was more influential at the decadal scale.

With the aim of highlighting the importance of taxonomical groups for TB dynamics, we analysed the partitioning variance of a model having the taxonomical groups as explanatory variables, *i.e*. TB = [CHLO] + [CRY] + [DIAT] + [DINO] + [AEM].

While the pure variance explained by these groups made up 53% of overall variability, AEM without interaction with TGBs did not explain any variance (Fig. 5b). The interaction of TGBs and AEM periodicity amounted to around 15%. MCA revealed that all TGBs, except Cryptophytes, were important for TB at the annual scale. However, the variability of Cryptophyte biomass was remarkable at the decadal scale (see Supplementary Table S2).

**Figure 6:**
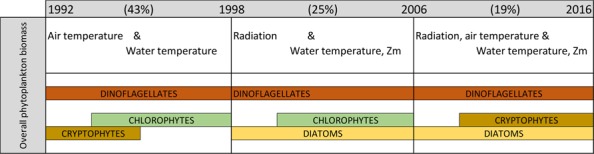
Diagram relating environmental variables that significantly control the biomass of phytoplankton groups in Las Madres lake in each period. The maximum adjusted variance explaining the biomass of each interval is shown in brackets (see also Table 4). Positions and thickness of each TG band are related with variabilities explained by environmental factors in each period. Zm, upper mixed layer.

When the whole series was split into the three already-envisaged periods (see above), air and water temperature explained TB in the first period (1992–1998), also explaining the biomass time course of the taxonomical groups (Chlorophytes, Cryptophytes and Dinoflagellates, Table 4, Fig. 6). In addition, other physical variables, such as underwater light climate and the upper mixed layer, controlled Chlorophyte and Dinoflagellate biomass, respectively. Water chemistry did not explain any variability of either TB or TGBs. Diatom biomass did not appear to be controlled by any factor in this first period.

**Table 4:** Control variables that explained more than 5% of total phytoplankton biomass or taxonomical group biomass with their percentage of explained variance (R^2^).

Response variable	VARIABLES R2 >5%		MODELS		
	Control variable	R^2^	R^2^adj of selected variables	Environmental factor	R^2^adj
1992–1998					
Total Biomass	Air temperature	41	41 (a)	LOC	41
	Water temperature	40	40 (a)	PHY	40
Chlorophyte biomass	Air temperature	33	33 (a)	LOC	37
	Radiation	16	4 (a)		
	Transparency	18	11 (a)	PHY	43
	Water temperature	32	32 (a)		
Cryptophyte biomass	Air temperature	18	18	LOC	18
	Water temperature	20	20	PHY	20
Dinoflagellate biomass	Air temperature	43	43	LOC	43
	Radiation	40			
	Zm	26		PHY	38
	Water temperature	38	38		
1998–2006					
Total Biomass	Air temperature	18		LOC	25
	Radiation	25	25		
	Zm	18		PHY	20
	Water temperature	20	20		
Chlorophyte biomass	Air temperature	18		LOC	19
	Radiation	19	19		
	Water temperature	15	15	PHY	15
Diatom biomass	Radiation	6	6	LOC	6
	Zm	8	8	PHY	8
Dinoflagellate biomass	Air temperature	23		LOC	24
	Radiation	24	24		
	Zm	12		PHY	17
	Water temperature	17	17		
2006–2016					
Total Biomass	Air temperature	14		LOC	19
	Radiation	19	19		
	Zm	11		PHY	15
	Water temperature	15	15		
Cryptophyte biomass	Air temperature	5		LOC	9
	Radiation	9	9		
	Water temperature	6	6	PHY	6
Diatom biomass	Air temperature	5		LOC	7
	Radiation	7	7		
	Water temperature	7	7	PHY	7
Dinoflagellate biomass	Air temperature	5	2	LOC	14
	Radiation	12	12		
	Zm	7	7	PHY	7
	Water temperature	6			

Also included, their adjusted explained variance (%R^2^_adj_) if selected by forward selection procedure and the adjusted explained variance of the individual effects of factors (last column), without interactions. This last value results from the RDA and variance partition analysis. Analysis performed for three consecutive periods occurring in Las Madres lake for 1992–2016. Probability of all variances was lower than 0.001. Abbreviations as in Table 3. For more information on statistical tools see Supplementary methods.

Local climate (solar radiation; R^2^ = 25%) and lake physics (water temperature; R^2^ = 20%) controlled TB during the second period (1998–2006, Table 4, Fig. 6). Solar radiation was the variable that best explained Chlorophyte, Diatom and Dinoflagellate biomass, with the upper mixed layer and water temperature also explaining some variability, albeit with an opposite sign. Cryptophyte biomass was not explained by any variable tested in this period (Table 4).

Finally, from 2006 to 2016 a decreasing variance, not greater than 19% of overall variability, was explained by environmental factors. Again, local climate and water physics jointly explained TB and TGBs (Table 4, Fig. 6). Solar radiation and water temperature were also important for Cryptophyte- and Diatom biomass, albeit less than for TB, and the upper mixed layer was also a key variable for Dinoflagellate biomass (Table 4). No regional teleconnections explained any statistically significant variability in any of the periods tested (P > 0.05).

## Discussion

In Las Madres lake, where no anthropogenic stress other than global warming has seemingly occurred^31^, there have been strong changes in phytoplankton biomass over the last 25 years. In accordance with our first three hypotheses, the total biomass of phytoplankton and those of their taxonomical groups exhibit trends and several periodicities which do not match entirely across those levels of organization and time periods (Table 2). TB experienced a reduction in the steepness of long-term trend over time, and its variability was strongly dependent upon scale; both facts occurred because TB drivers changed along with time scales, from months to decades. This fact was previously demonstrated but on key abiotic elements of aquatic environments (*e.g*. dissolved oxygen, nutrients)^2,32^. Taxonomical groups presented periodicities from intra-annual (*e.g*. Chlorophytes) to decadal (Cryptophytes). These different dynamics crystallize in changes in taxonomic composition that result in three very different periods (6, 8 and 10 years) throughout the time series studied. Thus, TB trends and periodicities are more related to one or another taxonomic group depending on the analysed period (Table 2).

These changes have arisen as a response to the local abiotic environment (Tables 3–4, Figs 5–6). Our study shows that phytoplankton is a complex assemblage whose elements relate to different controlling factors at different temporal scales, as we suggested in our fourth hypothesis. Therefore, TB dynamics is the emergent result of the relationship “phytoplankton groups-abiotic controlling factors-periodicities” and of the considerable variability of these relationships throughout the series (Fig. 6). We have evidenced that phytoplankton is sensitive to temperature throughout the whole monthly series (Tables 3–4). Moreover, we can suggest that warming, as annual averaged of air- and water temperature is taking place (see Supplementary Fig. S2a). However, such a temperature increase does not result in phytoplankton growth, but rather in a decrease in TB, because a higher temperature does not imply more TB since the effects of a temperature increase are complex in lakes^11^. An example of the latter is that a positive effect of a temperature increase on phytoplankton growth could be counterbalanced by another effect of temperature, such as a reduction in the mixing layer (see Supplementary Fig. S2b), which can potentially reduce the necessary nutrient availability for primary production during water column stratification^12^. This explanation, for the case in question, would be supported by the negative relationship of TB with the upper mixed layer, since spring algal growth occurs during early stratification after the winter mixing in Las Madres lake. Another process that may be occurring at the same time, and which would explain the negative relationship of warming with phytoplankton biomass, is the gradual disappearance of the effects of the internal nutrient dynamics in a newly-formed lake. This process, which that usually occurs over years in recently-built freshwater ecosystems such as reservoirs^23,33^, reduces the amount and variability of TB as we have observed (Fig. 1).

**Figure 7:**
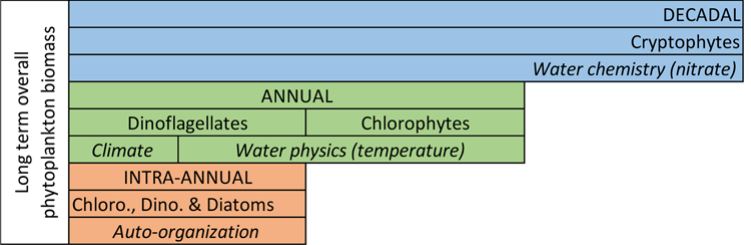
Diagram depicting the most relevant periodicities experienced by each taxonomical group and the sets of environmental variables likely controlling them over 25 years in Las Madres lake.

TB dynamics over the whole period of study showed three main types of periodicity: decadal, annual and intra-annual. The decadal change in TB is the result of the decadal change in the taxonomic group of Cryptophytes, whose likely controlling factor is related with nitrate (Fig. 7). This long-term response to nitrate is very interesting, albeit hard to explain. Cryptophytes are known to uptake ammonia preferably as a nitrogen source^34^. Since they are known to perform migrations in the water column of lakes^35^, they might obtain this compound from the bacterial reduction of nitrate in the anoxic conditions that prevail in Las Madres hypolimnion in summer. Such conditions might be enhanced by the, already mentioned, weakening of water-column mixing.

Inter-annual variability of phytoplankton composition showing three different states, and the existence of different periodicities in the signals, encouraged us to analyse, separately for each time period, the relationships of TB and TGBs with the controlling factors. Annual cycles during the earlier studied years showed higher variability, a fact reflected by both the wavelet analysis and the increasingly lower explained variance of annual periodicity (Table 2, Fig. 7). AEM and normalized wavelet analyses of the overall series failed to detect annual periodicities for taxonomical groups that could be otherwise identified when these analyses were applied to each interval (Table 2). The reason for this is that the amplitude of periods also decreased due to the decreasing TB trend over 25 years, another remarkable fact at these time intervals. The higher average value of the signal was associated with a stronger seasonality, compared to what occurred when biomass averages diminished. This lack of stationarity in the amplitude of the short time scale would explain why both analyses (*i.e*. AEM and normalized wavelet) enabled us to detect these patterns only during the first time interval. Therefore, when partitioning the time series, the amplitude of annual periods is less variable in the resulting time interval, allowing its detection with AEM and wavelet analysis. Thus, the division of the whole series into periods is a good recommendation for future works that aim to disentangle the drivers of biotic dynamics. As a result new periodicities would emerge, allowing to observe, for example, the annual scale previously undetected for some taxonomical groups as Cryptophytes and Diatoms (Table 2).

The dominant mode of TB variability in Las Madres lake is the annual mode (Table 2). Our study gives more weight to local climate (solar radiation, air temperature) and water physics (water temperature) as controlling factors of biomass (Tables 3–4, Fig. 4). Virtually all groups of algae respond to solar radiation and temperature, thus demonstrating an expected seasonality (*i.e*. annual periodicity). Fundamentally Dinoflagellates and, to a lesser extent, Chlorophytes are the taxonomic groups most related with these physical factors, both groups being considered as characteristic of the period of summer stratification in temperate lakes^16^. In addition, they are the two dominant groups in the third period of study (2006–2016), when the increase in temperature and its already mentioned consequences (for example, the weakening of water-column mixing) occur.

The intra-annual bimodal pattern (six months of periodicity) of TB, commonly expressed as the spring and autumn phytoplankton blooms, represents an alternation of periods of stratification and mixing. This alternation is key for the dynamics of motile (*i.e*. Dinoflagellate) and non-motile (*i.e*. Diatoms) phytoplankton species, respectively^16^. Both are precisely the taxonomic groups that have been found to be related with mixing depth and intra-annual periodicity of species substitutions in Las Madres lake during the first two periods, when the whole series was partitioned. The intra-annual bimodal pattern disappeared in the last interval of the series, giving way to the unimodal model of a TB peak in spring/summer. As mentioned earlier, thermal changes imply changes in water-column stratification, and it is well known that warming results in stronger stability of the upper mixed layer, thus enabling Dinoflagellates (the only group inversely related to the upper mixed layer in the latter period) to increase over an ongoing longer stratification^36^.

However, some variability of the studied series remains unexplained. Biotic variables important for phytoplankton, such as bacteria and zooplankton^16,37^, are poorly related to TB and TGBs at the monthly scale in Las Madres lake (Alvarez-Cobelas *et al*., unpublished results), maybe because they are relevant at shorter scales than those used in this study.

Another process that could partially explain TB dynamics, and which is not dependent on the abiotic environment, is phytoplankton autogenic succession which commonly runs during the period of thermal stratification when physical stability of the water column remains relatively constant^16,23,38^. Throughout the 25 years, whenever lake warming reduces the intensity of the autumn-winter mixing period and hence extends the water-column stratification period, a longer auto-organizational period within the year would thus be permitted^16^. It is in this period that phytoplankton competition, and the ability of its species to distribute themselves in different layers of the water column, would explain the substitution of characteristic groups of phytoplankton species over time^16,20,38^. This lack of external drivers would agree with the gradual loss of TB variability attributable to the set of factors and periodicities analysed. Therefore, auto-organization remains as another likely, partial explanation for long-term phytoplankton dynamics.

Having monthly time series of the control variables of phytoplankton and its taxonomic groups over a 25-year period has allowed us to verify that the explanation for its long-term dynamics can be disentangled when we examine the response of taxonomical groups and their relationship with the environment. It also allows us to understand that responses occur at different scales. They are not stationary, but can be due to different agents and scales over time. This is the method that must be followed in order to explain the maximum long-term variance of phytoplankton, or of any other ecological group. In addition, our results show that the dynamics depends on several time scales (intra-annual to decadal); thus, it would be acceptable that part of the variance not yet explained could be understood if we had series with higher sampling frequencies (hours, days, weeks). Higher frequencies are related, for example, to metabolic processes or interactions between aquatic ecosystem groups (predation, competition with bacteria). Finally, it is necessary to consider whether the development of eco-evolutionary studies, which link evolutionary change and higher-level ecological variables, such as community composition, could explain the response of species and communities to long-term changes (such as global warming^39^).

To conclude, the dynamics of long-term phytoplankton biomass appears to be an emergent feature of all the aforementioned processes, also depending upon the time scale involved. Two features of long-term ecological dynamics are demonstrated by our study: 1^st^) some drivers of long-term lake dynamics can remain hidden, and can only be disentangled by a holistic approach; and 2^nd^) different levels of community organization, such as total biomass and biomass of taxonomical groups, are helpful to gain insights into the long-term patterns and processes involved. Our methodological approach has provided scientific knowledge on trends, periodicities and controlling factors for different levels of phytoplankton organization and multiscale changes in a warm, temperate oligo-mesotrophic lake. Such an approach could be profitably applied to the study of all these features and processes occurring in the long-term phytoplankton dynamics of lakes worldwide. Up to now, these responses had been depicted as (smoothing) trends, either along the oligo-eutrophication axis (or its reversal) or the global warming axis (*i.e*. stronger thermal effects on lake features over time), but some recent reports have shown that all these effects partly cancelled each other out (see Table 1). It is time to open the gate for more complex views that encompass phenomena, periodicities, interactions and controls previously overlooked in long-term ecology.

## Material and Methods

### Study site

Las Madres Lake is a seepage, oligo-mesotrophic, gravel-pit lake in an alluvial plain close to Madrid (semi-arid Mediterranean region, Central Spain, 40°18′N3°31′W), with a surface area of 3.4 Ha, and an average and maximum depth of 8 m and 19 m, respectively^40^. As a new environment created in the seventies, sand and gravel mining was abandoned in 1984. Since then, the lake has been a closed, largely wind-sheltered environment whose water inputs come from the underlying aquifer and rainfall. During winter its water-column temperature has rarely been found below 8 °C. More details on the hydrological, physico-chemical features of this lake and its biota are offered in Supplementary Information.

### Climate variables, lake sampling and laboratory procedures

Teleconnection variables (NAO, AO, EA and ENSO), compiled by the Climate Prediction Centre of the National Oceanic and Atmospheric Administration, were gathered from http://www.cpc.ncep.noaa.gov/. Local climate variables were recorded at 10 min intervals by a meteorological station located 3 km from Las Madres lake. These variables were air temperature (°C) and incoming solar radiation (W/m^2^); their monthly averages were calculated for this study.

The lake was sampled monthly from January 1992 to December 2016, between 10:00 and 12:00 (GMT) at a 12 m deep station located in the central area of the lake (see Supplementary Table S1). Physical water-column variables, such as temperature and radiation, were measured with different YSI and LI-COR probes, which were mounted on an SBE-19 Seabird rack from 2006 onwards. Local lake physical variables were: water transparency (Secchi disk depth, m), temperature (°C) and upper mixed layer (Z_m_, m). Water for chemical analyses and phytoplankton for this study was collected with a 5 L Niskin PVC bottle at every meter throughout the whole water column. Chemical variables were nitrate, ammonia (mg N/L) and total phosphorus concentrations (mg P/L). They were measured within half an hour after sampling following standard procedures^41^ up to December 2008 using classical spectrophotometry, and later employing a Seal-3 QuAAtro AQ2 auto-analyzer. Careful checks and intercalibrations were performed between techniques undertaken before and after that date to ensure that chemical data are comparable and all series were internally consistent. Silica and DOC data were discarded as variables for further analyses because of their usually high concentration in the lake (see Supplementary Table S1). Most long-term data for Las Madres lake are stored at http://www.sanchezandalvarezlab.es. For statistical analyses, abiotic factors were water-column averaged.

Phytoplankton samples were retrieved at each meter and immediately pooled for the whole water column, thus providing a single sample for each monthly datum. Biomass estimations and their conversion to carbon were calculated following standard methods. Supplementary Information shows more detailed information concerning the methods used to study phytoplankton. Phytoplankton taxonomical groups attaining up to 95% of overall biomass in each sample were selected for further analysis; these were Chlorophytes (CHL), Cryptophytes (CRY), Diatoms (DIA) and Dinoflagellates (DINO).

### Statistical analyses

We use methods implemented to quantify the relative importance of patterns and processes across time scales of ecosystems^24^: Asymmetric Eigenvector Maps^25^, variance partitioning^26^ and codependence analysis^27^. Moreover, because ecological communities can change over time and their time series can be non-stationary a fourth method must be taken into account: wavelet analysis^28^. This tool enables the emergence and/or disappearance of periodicities throughout the whole series to be identified^28^.

#### Spectral analysis of long term series

Time series were standardized and detrended^24^. We applied Asymmetric Eigenvectors Maps analyses (AEM) to obtain a set of variables that represents periods with decreasing time scales. For example, these variables represent intra-annual periodicity, annual periodicity, and seasonal and monthly periodicity^42^. Both the monthly sampling and the 25 years of data enabled us to search for: i) intra-annual periodicities longer than a month; ii) annual periodicities; iii) inter-annual periodicities of more than a few years, and iv) decadal periodicities when the cycle is longer than ten years. Variables implying different periodicities were uncorrelated to each other, and could be used as covariates to model phytoplankton responses to environmental variables^29^.

AEM spectral decomposition is well suited for a time series in which periodicities are constant over time, but is unable to characterize non-stationary series, whose main periodicities change over time. Therefore, we also used wavelet analysis which reveals how different scales (periodic components) of the time series emerge and disappear over time^28^.

#### Controlling factors of phytoplankton dynamics

The target variables (response variables) were: the time series of total phytoplankton biomass (TB hereafter) and the biomass of the main taxonomical groups (TGBs). The five factors that could predict the dynamics of the response variables are five matrices constructed with variables from the set of variables listed in the previous section, selected by applying forward selection with two stopping criteria following^43^. The variables of regional climate (*i.e*. teleconnection indices such as NAO, ENSO, etc…) are included in factor [REG], local climate in [LOC], lake physics in [PHY] and lake chemistry in [CHE], as well as the matrix of periodicities [AEM].

Multivariate Redundancy Analysis (RDA) was used to establish the effect of the five predictive factors on phytoplankton dynamics (*i.e*. TB and TGBs). Variance partitioning reveals how much variance (R^2^adj) of target biotic variables is explained by pure individual effects of each factor and how much is explained by their interactions^24,26^. Clearly, this procedure produces much lower values of explained variability than other more commonly employed approaches, such as plain correlation and its derivatives, but this is because temporal components (such as trend and periodicities) and covariation of variables are studied separately. In addition, co-dependency analysis was applied to evaluate which environmental variable was relevant for each phytoplankton periodicity^27^.

#### Time series partitioning

Since our hypothesis is that there could be different dynamic periods over the whole long-term study, and changes in taxonomical compositions are expected at longer scales, samples were clustered with the UPGMA (unweighted pair group algorithm) applied to the Bray-Curtis similarity matrix calculated on yearly-averaged biomass fractions (%) of the main taxonomic groups. Random bootstrap permutations (999) tested the relevance of each node and a constrained function ordered dates.

A flowchart of statistical methodologies used in this study related to the topics they intend to solve, plus complementary information on statistical analyses presented in this section and the software used, are reported in Supplementary Information.

## Supplementary information

Supplementary material

## Data Availability

The datasets generated during and/or analysed during the current study are available in the http://www.sanchezandalvarezlab.es repository.
