# Supplementary material for: Long-Term Phytoplankton Dynamics in a Complex Temporal Realm
Source: Sci Rep. 2019 Nov 4;9:15967. doi: 10.1038/s41598-019-52333-z (PMC6828802; doi:10.1038/s41598-019-52333-z)
Supplement: Supplementary file 1 — Supplementary material [file 41598_2019_52333_MOESM1_ESM.pdf]

LONG-TERM PHYTOPLANKTON DYNAMICS IN A COMPLEX TEMPORAL REALM

Miguel Alvarez-Cobelas, Carmen Rojo & José Benavent-Corai

Index

1. *Supplementary information on methods* pages 1-6
2. *Supplementary figures* pages 7-8
3. *Supplementary tables* pages 9-10

1. Supplementary information on methods

Study site

The lake becomes anoxic in summer at bottom layers as a result of a decaying littoral, emergent macrophytic biomass which entered the previous autumn. The lake is usually P-limited, oligo-mesotrophic (Table S1) and it has a medium dissolved organic carbon content, but it occasionally reaches high values of variables like inorganic nitrogen and water colour because of a vernal upwelling of hypolimnetic water, triggered by inputs of warmer seepage water. Regional teleconnection effects on local climate are weak in Las Madres lake (Hernández *et al.* 2015).

Phytoplankton biomass was dominated by Diatoms, Cryptophytes and Dinoflagellates, the main species being *Cyclotella ocellata*, *Cryptomonas erosa* and *Peridinium willei*. Changes in zooplankton composition have been negligible in the long-term, its assemblage being dominated by the copepods *Arctodiaptomus salinus* and *Tropocyclops prasinus* and the cladoceran *Daphnia longispina*, with a court of smaller cladocerans, and up to 50 species of ciliates and rotifers, whose biomass was usually much lower than those of larger crustaceans.

Plankton was fixed in the field with iodine-lugol for phytoplankton. Its counting was undertaken following Rott (1981), with 400 specimens of the main species being counted in each sample under an inverted microscope, and their classification being carried out following standard taxonomic texts. Biomass estimations were calculated following Rott (1981). Biomass data were given in carbon, following standard conversion factors (Reynolds 2006).

Statistical analyses

1. What are the trend and periodicities of phytoplankton biomass?

AEM and Forward Selection analyses.- In a first step, we applied Asymmetric Eigenvector Maps analyses (AEM) that summarized the temporal structure of sampling design in a set of variables that represents periods with decreasing time scales (Blanchet *et al.* 2014). Periodicities were uncorrelated to each other, and could be used as covariates to model phytoplankton responses to environment variables (Baho *et al.* 2015). Both the monthly sampling and the 25 years of data enabled us to search for i) intra-annual periodicities longer than a month, ii) annual periodicities, iii) inter-annual periodicities for more than a few years, and iv) decadal ones when the cycle is longer than ten years, also called "interdecadal". In a second step, we selected trend and periodicities (predictor variables) that best fitted to each phytoplankton time series analysed (*i.e.* response variable, namely total phytoplankton biomass and biomass of each taxonomical group) by applying forward selection methodology with two stopping criteria (see our Table 2).

2. Does phytoplankton dynamics show the same periodicities over the whole time series?

We have used **wavelet analysis** to perform the temporal decomposition of the signal by estimating its spectral features as a function of time (Torrence and Compo 1998). Once phytoplankton dynamics have been standardized and detrended, this approach reveals how different scales (periodic components) of the time series emerge and disappear over time since the wavelet function is stretched in time by varying its scale. This procedure has already been used successfully in ecology, and more specifically in plankton analysis (Winder and Cloern 2010). The relevance of each scale is represented by a normalized heat map, where different periodicities are ranked in a vertical axis, and their emergence and disappearance can be noticed along a temporal axis (Torrence and Compo 1998; Liu *et al.* 2007). A red cone delimits the space outside of which spectral information must be interpreted with caution. Boundaries of regions with significant periods were limited with a black line after testing through Monte Carlo randomization by comparing the signal of interest with coefficients from a red-noise power spectrum (Torrence and Compo 1998). Out of the many different wavelet techniques available to build such a map, we have chosen the Morlet function which seems to be the most appropriate for phytoplankton time series and shows a good equilibrium between time and resolution scale (Winder and Cloern 2010). (see our Figures 1b, 3 and S1).

3. Are biomass responses of taxonomical groups different from those of total phytoplankton biomass?

To answer this, the description of trend and periodicities of total biomass (TB hereafter) and biomass of each taxonomical group (TGBs hereafter) at different time scales was undertaken by wavelet analysis in order to highlight the likely differences among them.

4. What are the controlling predictors of phytoplankton dynamics for each environmental factor?

Forward Selection with two stopping criteria.- Once standardized and detrended, the response variables were the time series of TB and TGBs. The environmental variables measured were standardized, detrended and grouped in four sets of environmental factors: regional climate [REG], local climate [LOC], lake physics [PHY] and lake chemistry [CHE]. Only those explaining at least 5% of phytoplankton variance were considered for this analysis, as they had a significance of biological relevance for making inferences; the remaining variables were discarded (see our Tables 3 and 4). Within each factor, we selected the variables that best explained phytoplankton responses by applying forward selection with two stopping criteria to prevent i) collinearity among predictors, ii) error I type inflation, and iii) the overestimation of the amount of explained variance (Blanchet *et al.* 2008). The selection of variables was carried out separately for each factor in order to quantify their individual importance in further analyses. The amount of explained variance by each selected predictor variable was estimated with the adjusted R^2 statistic (R^2adj), which provides unbiased estimates. Its statistical significance was evaluated with 999 Monte Carlo permutations (Peres-Neto *et al.* 2006) (see our Tables 3 and 4). When no predictive variables were selected within a given environmental set of factors, it was excluded for remaining analyses.

Codependence analysis was later applied to assess the relevance of each selected abiotic predictor for each phytoplankton periodicity. Its statistical significance was assessed with the absolute τ statistic (absτv), whereas the p-value (Pvalfam) was estimated by independent permutations (Guénard *et al.* 2010). (See our Table S2).

5. What is the relative importance of controlling factors?

RDA and Variance partitioning.- Multivariate Redundancy Analysis (RDA) was used to model the effect of environmental sets of factors (matrices of selected predictive variables) on

phytoplankton dynamics (*i.e.* TB and TGBs). This model also included a temporal matrix called *AEM* which grouped the selected periodicities from previous Asymmetric Eigenvector Maps analyses. Variance partitioning provides R^2_{adj} of pure individual effects of each factor and their interactions (Peres-Neto *et al.* 2006). The pure effect of abiotic factors was tested by permuting residuals of the reduced model. However, the explained variances arising from interaction of factors cannot be tested (Legendre and Legendre 2012). This procedure produces much lower fractions of variability in the signal explained by its controlling factors than other more commonly employed approaches, such as plain correlation and its derivatives, but this is because temporal components (such as trend and periodicities) and covariation of variables are studied separately (see our Tables 2 and 3).

6. Would these controlling factors be the same across all organization levels and/or throughout the whole period of study?

We have repeated RDA and variance partitioning with TGBs (see our Table 3) to compare results with those of TB.

In addition, are changes in taxonomic composition indicating different compositional periods over the whole time series?

Clusters analysis of monthly phytoplankton samples calculated on yearly-averaged biomass fractions (%) of the main taxonomic groups, plus the shape of TB dynamics and patterns of % of TGBs, enabled us to discriminate three time periods throughout the long-term series (*i.e.* 1992-1998, 1999-2005 and 2006-2016). Hence all the aforementioned analyses (points 1,2,3,4) were performed again for each period and we compared both the resulting figures from wavelet analysis and tables and those resulting from AEM analysis among the different periods in order to see if phytoplankton dynamics differed throughout the time period of study (see our Table 4).

Statistical Software.- Asymmetric Eigenvectors Maps analyses along with Moran tests were undertaken with the "aem.time" function of the "AEM" package, implemented in R 2.15.1 statistical software (Blanchet et al. 2014; R Development Core Team 2015). Wavelet analysis was done using functions by Torrence and Compo (1998) compiled in

http://atoc.colorado.edu/resaerch/wavelets/ and modified to include corrections by Liu et al. (2007). Wavelet analysis, forward selections with double stopping criteria, redundancy analysis, variance partitioning and codependence analysis were performed with scripts in the Matlab language of technical computing (MATLAB version 8.5.0., MathWorks). The clusters used to split the whole time of study into shorter, distinct stretches and cross-correlation analyses were calculated using the PAST package (Hammer et al. 2001).

References

Blanchet, F.G., P. Legendre, and D. Borcard. 2008. Forward selection of explanatory variables. Ecology **89**: 2623–2632. doi:10.2307/1268227.

Blanchet, F.G., P. Legendre, and O. Gauthier. 2014. AEM: Tools to construct Asymmetric eigenvector maps (AEM) spatial variables. R package version 0.5-2/r119. https://R-Forge.R-project.org/projects/sedar/

Guénard, G., P. Legendre, D. Boisclair, and M. Bilodeau. 2010. Multiscale codependence analysis: an integrated approach to analyze relationships across scales. Ecology **91**: 2952–2964. doi:10.1890/09-0460.1.

Hammer, Ø., D.A.T. Harper, and P.D. Ryan, 2001. PAST: Paleontological Statistics Software Package for Education and Data Analysis. Palaeontologia Electronica **4**(1): 9 pp.

Hernández, A., R.M. Trigo, S. Pla-Rabes, B. Valero-Garcés, B., S. Jerez, M. Rico-Herrero, J.C. Vega, M. Jambrina-Enríquez, and S. Giralt. 2015. Sensitivity of two Iberian lakes to North Atlantic circulation modes. Climate Dynamics doi:10.1007/s00382-015-2547-8.

Legendre, P., and L. Legendre. 2012. Numerical Ecology. 3rd edition. Elsevier, Dordrecht.

Liu, Y., X.S. Liang, and R.H. Weisberg. 2007. Rectification of the bias in the wavelet power spectrum. J. Atmosph. Ocean Technol. **24**: 2093-2102. doi:10.1175/2007JTECHO511.1.

Peres-Neto, P.R., P. Legendre, S. Dray, and D. Borcard. 2006. Variation partitioning of species data matrices: estimation and comparison of fractions. Ecology **87**: 2614–2625. doi:10.1890/0012-9658(2006)87[2614:VPOSDM]2.0.CO;2.

Porter, K.G., and Y.S. Feig. 1980. The use of DAPI for identifying and counting aquatic microflora. Limnol. Oceanogr. **25**: 943-948. doi:10.4319/lo.1980.25.5.0943.

R Development Core Team. 2015. R: a language and environment for statistical computing. R Foundation for Statistical Computing, Vienna, Austria.

Reynolds, C.S. 2006. Ecology of Phytoplankton. Cambridge University Press. Cambridge (UK).

Rott, E. 1981. Some results from phytoplankton counting intercalibrations. Schweiz. Z. Hydrol. **43**: 34-62.

Torrence, C., and G.P. Compo. 1998. A practical guide to wavelet analysis. Bull. Am. Meteorol. Soc. **79**: 61-78. doi: 10.1175/1520-0477(1998)079<0061:APGTWA>2.0.CO;2.

Winder, M., and J.E. Cloern. 2010. The annual cycles of phytoplankton biomass. Phil. Trans. R. Soc. B **365**: 3215–3226. doi:10.1098/rstb.2010.0125.

Flowchart of statistical methodologies used in this study and the topics they intend to solve. AO: Atlantic Oscillation; CHL: Chlorophyte biomass; CRY: Cryptophyte biomass; DIA: Diatom biomass; DINO: Dinoflagellate biomass; EA: Eastern Atlantic Oscillation; ENSO: El Niño Southern Oscillation; NAO: Northern Atlantic Oscillation, Secc: Secchi transparency, TP: Total phosphorus.

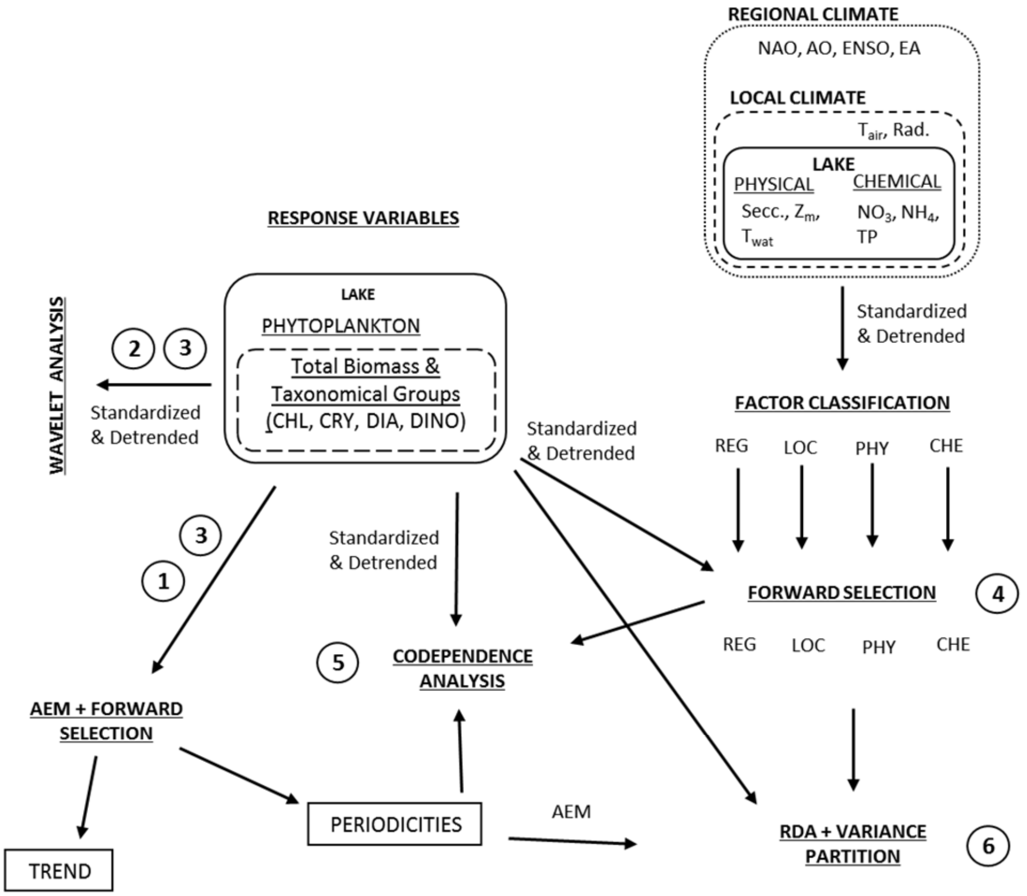

1. *What are the trend and periodicities of phytoplankton biomass?*
2. *Does phytoplankton dynamics show the same periodicities over the whole time series?*
3. *Are biomass responses of taxonomical groups different from those of total phytoplankton biomass?*
4. *What are the controlling predictors of phytoplankton dynamics for each environmental factor?*
5. *What is the relative importance of controlling factors?*
6. *Would these controlling factors be the same across all organization levels and/or throughout the whole period of study?*

In addition, are changes in taxonomic composition indicating different compositional periods over the whole time series? The aforementioned analyses (points 1,2,3,4) were performed again for each period.

2. *Supplementary figures*

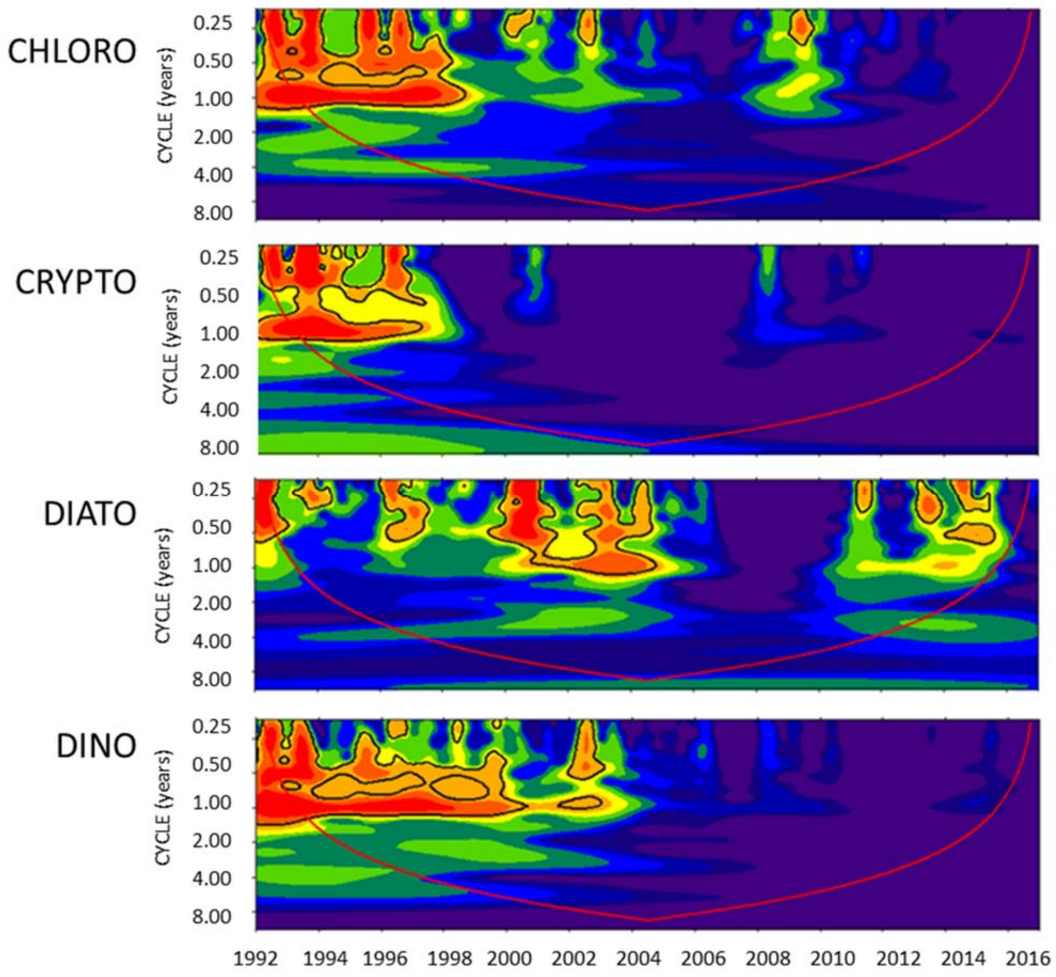

Figure S1. Continuous wavelet power spectra showing the biomass periodicity of the main taxonomic groups in Las Madres lake from 1992 to 2016. The thick black contour delimits the significant periodicities ($P < 0.05$) and the red line denotes the cone of influence, where edge effects may distort the interpretation of that region; colours reflect the power intensity (dark red indicates high power; dark blue indicates low power).

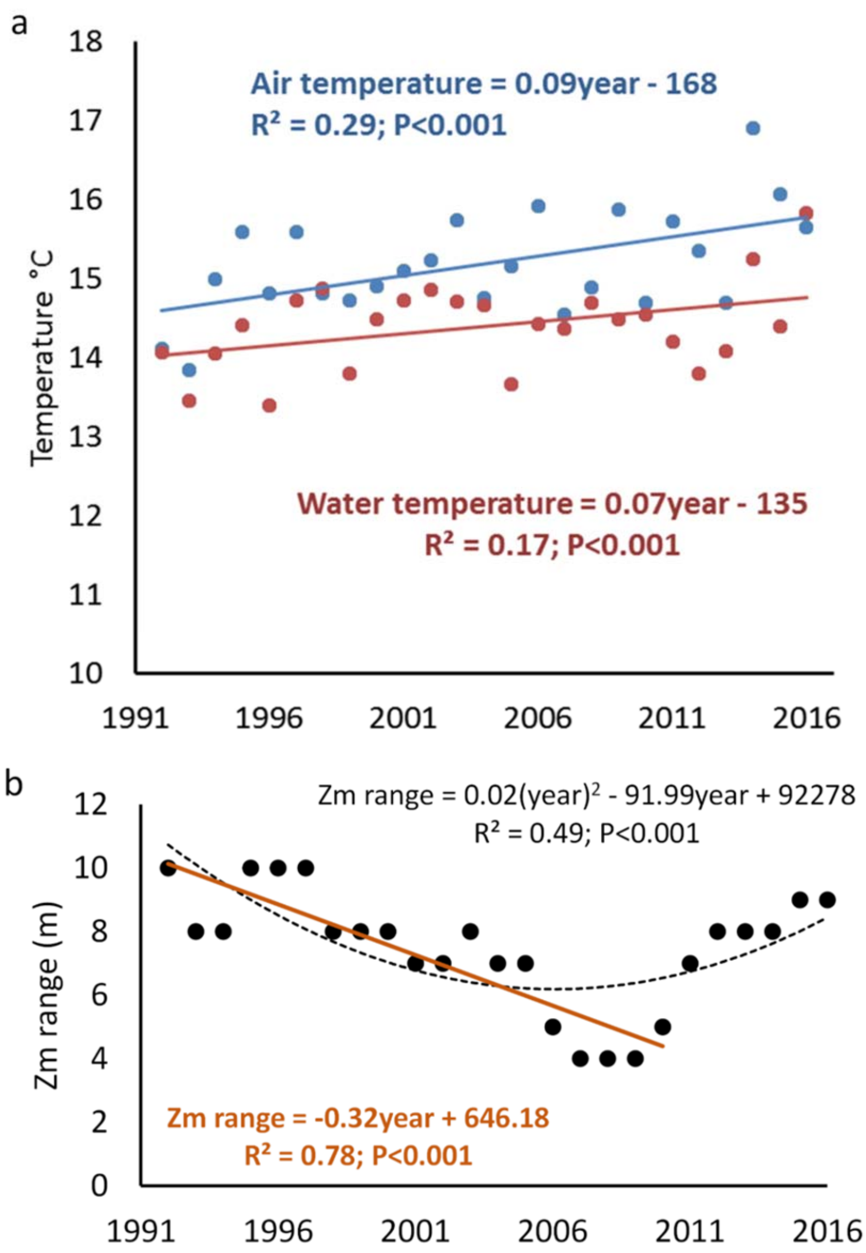

Figure S2. a) Long-term annual averages of air- and water column-averaged temperatures in Las Madres lake. Reduced Major Axis regressions are shown jointly with their variance (R^2) and probability (P). b) Long-term range of the upper mixed layer, this range being the difference between the highest and the lowest depth of the upper mixed layer within each year. These values have been fitted to a polynomial function for the whole time series (1992-2016; black dashed line, function in black) and to a simple linear function for the first 20 years of study (1992-2011; brown line and function).

3. Supplementary tables

Table S1. Ranges of variables recorded in the water column of Las Madres lake for 1992-2016.

Variable	Range
Water temperature (ºC)	6.4-28.2
Secchi transparency (m)	0.3-7.2
PAR attenuation (1/m)	0.3-4.6
Dissolved oxygen (mg/L)	0.0-11.0
Conductivity (µS/cm)	1400-3500
pH	6.06-8.41
Alkalinity (meq/L)	3.4-7.9
DOC (mg C/L)	3.1-24.9
Suspended matter (mg/L)	0.5-84.0
Silica (mg Si/L)	0.59-13.11
Sulfate (meq/L)	2.2-45.8
Chloride (meq/L)	6.7-7.9
Calcium (meq/L)	8.0-13.1
Magnesium (meq/L)	8.0-8.3
Sodium (meq/L)	9.2-11.3
Potasium (meq/L)	0.4-0.6
Nitrate (mg/L)	0.00-27.40
Ammonia (mg/L)	0.000-2.868
Total nitrogen (mg N/L)	0.12-8.46
SRP (mg P/L)	0.000-0.218
Total phosphorus (mg P/L)	0.000-1.300
Chlorophyll-a (µg/L)	0.1-10.0
Bacterial biomass (mg C/L)	0.0065-0.1004
Phytoplankton biomass (mg C/L)	0.0002-2.2075
Zooplankton biomass (mg C/L)	0.0001-0.1700

Table S2. Factors controlling overall phytoplankton periodicity in Las Madres lake for 1992-2016, arising from co-dependence analysis.

**Variable**	**Periodicity**	**$_{abs}$Cy,x;w**
Air temperature	ANNUAL	116.3
Radiation	ANNUAL	123.5
Water temperature	ANNUAL	113.1
Zm	ANNUAL	95.0
Ammonia concentration	DECADAL	49.5
Chlorophyte biomass	ANNUAL	39.1
Cryptophyte biomass	DECADAL	44.5
Diatom biomass	ANNUAL	27.7
Dinoflagellate biomass	ANNUAL	57.4

For each periodicity, independent variables explaining overall biomass variability, along with the absolute value of co-dependence ($_{abs}$Cy,x;w), are shown. The statistic ($_{abs}\tau$v) and its significance ($Pval_{fam}$) for the standardized and detrended series were 1.01 and 0.002, respectively. Zm: depth of upper mixed layer.
